# The association between triglyceride glucose-body Mass Index and in vitro fertilization outcomes in women with polycystic ovary syndrome: a cohort study

**DOI:** 10.1186/s13048-024-01416-1

**Published:** 2024-04-26

**Authors:** Xin Li, Ting Luan, Yi Wei, JuanJuan Zhang, Chun Zhao, Xiufeng Ling

**Affiliations:** 1 Department of Reproductive Medicine, Women’s Hospital of Nanjing Medical University, Nanjing Women and Children’s Healthcare Hospital, 123 Tianfeixiang, Mochou Road, Qinhuai District, Nanjing, JiangSu China; 2 Department of Obstetrics and Gynecology, Women’s Hospital of Nanjing Medical University, Nanjing Women and Children’s Healthcare Hospital, Nanjing, China

**Keywords:** Assisted reproductive technology, Polycystic ovary syndrome, Triglyceride glucose-body mass index, In vitro fertilization outcomes

## Abstract

**Background:**

Polycystic Ovary Syndrome (PCOS) is a common reproductive disorder that frequently affects fertility. The TyG-BMI (Triglyceride glucose-body mass) index is a newly explored parameter that may be linked to reproductive results in individuals with PCOS. Nevertheless, its connection with outcomes in In Vitro Fertilization (IVF) procedures remains uncertain.

**Methods:**

This study included a total of 966 females who underwent IVF treatments for PCOS. At the baseline, the participants were categorized into four groups according to the quartiles of TyG-BMI measured prior to oocyte retrieval. Subsequently, the study compared the differences in clinical and laboratory outcomes among these four groups.

**Results:**

Patients in higher TyG-BMI quartiles exhibited a decreased number of retrieved oocytes, 2PN embryos, and available/high-quality embryos (*P* < 0.05 for Q1-Q4). Additionally, the multivariable regression analysis revealed that individuals in the top quartile of TyG-BMI had a lower count of accessible embryos (β = -0.224, *P* = 0.257) and a decreased number of high-quality embryos (β = -0.352, *P* = 0.028) in comparison to those in the lowest quartile. Nevertheless, there were no notable variances detected in the rates of pregnancy or live births among these quartiles. Furthermore, a linear correlation was noted between the TyG-BMI index and the quantity of accessible embryos (*P*-non-linear = 0.6, *P*-overall < 0.001), along with high-quality embryos (*P*-nonlinear = 0.026, *P*-overall = 0.006). In contrast, there was no notable linear correlation found between the TyG-BMI index and the available embryo rate (*P*-nonlinear = 0.60, *P*-overall = 0.8).

**Conclusions:**

The results of this research emphasize the notable correlation between TyG-BMI and IVF results in females diagnosed with PCOS. The interplay of insulin resistance and disorders of lipid metabolism may indeed play a pivotal role in influencing the assisted reproductive outcomes of patients with PCOS. Considering these findings, TyG-BMI proves to be a valuable indicator for exploring this potential association.

**Supplementary Information:**

The online version contains supplementary material available at 10.1186/s13048-024-01416-1.

## Background

Polycystic ovary syndrome (PCOS) is a major cause of female infertility, posing significant challenges to reproduction. Affecting a significant portion of women during their childbearing years, its prevalence is estimated to range from 6 to 20% [1, 2]. To diagnose PCOS according to the Rotterdam criteria, it is necessary to have at least two of the following symptoms: oligoanovulation, signs of hyperandrogenism (either clinical or biochemical), and the identification of polycystic ovaries using ultrasound [3], other endocrine disorders should be ruled out. Metabolic irregularities linked to PCOS can disrupt ovarian functionality and follicle growth, leading to diminished quality and quantity of oocytes, alongside lower rates of pregnancy and live births. The manifestation of PCOS symptoms varies considerably among individuals, and its precise cause remains unclear. According to recent research, the development of PCOS may have multiple causes, including oxidative stress caused by reactive oxygen species (ROS) [4], inflammatory responses [5], genetic factors [6], excessive embryonic androgen exposure [7], unhealthy lifestyle choices [8], and hormonal imbalances [9]. Furthermore, mounting evidence suggests that insulin resistance (IR) is a key factor in the development of PCOS, which is associated with an increased susceptibility to metabolic disorders like type 2 diabetes mellitus (T2DM), dyslipidemia, nonalcoholic fatty liver disease (NAFLD), and cardiovascular diseases (CVD) [10].

PCOS shares numerous pathophysiological traits with Metabolic syndrome (MetS), which is defined by a collection of metabolic dysfunctions such as central obesity, dyslipidemia, high blood pressure, and elevated blood sugar levels. Research indicates a higher prevalence of MetS among PCOS patients compared to the general population, underscoring the role of IR as a central component of both conditions [11]. Although the hyperinsulinemic-euglycemic clamp (HIEC) test is considered the most accurate method for evaluating insulin resistance, its extensive intricacy and high expenses restrict its widespread adoption. HIEC, in correlation with the homeostatic model assessment for IR (HOMA-IR), presents a viable alternative for conducting extensive studies [12]. Nevertheless, routine health examinations and clinical practices seldom measure insulin levels, making HOMA-IR data acquisition challenging [13]. The triglyceride-glucose (TyG) index, developed by South American researchers, has shown a strong correlation with both HIEC and HOMA-IR [14], presenting a more accessible and cost-effective method for evaluating IR.

Obesity, measured through body mass index (BMI), is another significant element linked with insulin resistance (IR) [15]. The TyG-BMI index, combining the TyG index and BMI, has demonstrated a close correlation with HOMA-IR in assessing IR in Korean and Chinese populations [16, 17]. This index has proven more effective than the TyG index alone in predicting metabolic diseases and cardiovascular disease (CVD) [18–20]. Nevertheless, the correlation between the TyG-BMI index and the outcomes of in vitro fertilization (IVF) treatment in women with PCOS has not been thoroughly investigated. Women with PCOS often rely on assisted reproductive technology (ART) for conception due to challenges in oocyte maturation and other fertility factors. The gonadotropin-releasing hormone antagonist (GnRH-ant) protocol has become increasingly popular, among individuals with PCOS due to its shorter duration of stimulation, decreased need for gonadotropins, and reduced risk of OHSS (ovarian hyperstimulation syndrome) [21]. Success rates of In Vitro Fertilization (IVF) in women with PCOS exhibit significant variability, which is affected by multiple factors such as the woman’s age, ovarian reserve health, stimulation protocol approach, embryo quality, and endometrial receptivity Several studies have investigated the role of IR, dyslipidemia, and obesity as potential predictors of IVF outcomes in women with PCOS, but the results are inconsistent and inconclusive [22, 23]. Moreover, most of these studies have used single or isolated measures of metabolic parameters, which may not reflect the complex interaction between IR and other metabolic factors. To address these concerns, our study aimed to investigate the relationship between the TyG-BMI index, measured prior to oocyte retrieval, and the effectiveness of assisted conception in PCOS patients undergoing treatment with the GnRH-ant protocol.

## Materials and methods

### Study design and participants

Conducted at Nanjing Medical University’s Women’s Hospital between January 2018 and September 2020, our study involved a cohort of participants and followed a hospital-based design. This study primarily examined PCOS patients experiencing their first IVF or ICSI (Intracytoplasmic sperm injection) embryo transfer (ET).To be eligible to participate, women between the ages of 20 and 40 with a diagnosis of PCOS based on the Rotterdam criteria [3], needed to meet at least two of the following conditions: experiencing irregular or absent ovulation, showing signs of hyperandrogenism either clinically or biochemically, and having polycystic ovaries. Other potential causes such as congenital adrenal hyperplasia, androgen-secreting neoplasms, or Cushing’s syndrome had to be ruled out. The utilization of either in vitro fertilization or intracytoplasmic sperm injection for the process of fertilization [3]. Following the GnRH-ant protocol for PCOS management. We excluded subjects for reasons such as: [1] Infertility causes unrelated to PCOS [2]. Previous ovarian surgeries or concurrent issues like endometriosis [3]. Disorders in liver, kidney, or thyroid function.Conditions that prevent ART or pregnancy include recurrent miscarriages (three or more losses), abnormalities in the uterus, chromosomal anomalies in parents, or any other condition that hinders the ability to conceive [5]. Male factor infertility (We defined male factor infertility as having one or more of the following abnormalities: sperm concentration < 15 million/ml, total sperm count < 39 million, progressive motility < 32%, normal morphology < 4%, or total motile sperm count < 9 million) [24].

### Ovarian stimulation protocol

A flexible GnRH-ant regimen was employed.From the second or third day of their menstrual cycle, the participants were given daily doses of 150–225 IU of recombinant FSH (rFSH, Gonal-F, Merck Serono, Italy). Once the dominant follicle exceeded 12–14 mm, the protocol was modified to incorporate a daily dose of 0.25 mg of GnRH-ant (Cetrorelix, Merck Serono, Darmstadt, Germany). Regular monitoring of follicular growth through ultrasound and hormone levels (FSH, LH, E2, P) enabled tailored adjustments to the Gn dosage. Once there were at least two follicles that measured more than 18 mm, a dose of hCG (Lizhu, China) of 10,000 IU was administered to facilitate oocyte maturation.Oocyte collection was scheduled 36 h post-hCG administration.A reduced hCG dose (5,000 IU) was utilized for ovulation induction in cases with a high OHSS risk.On ovulation trigger day, we assessed serum sex hormones and endometrial thickness.

### Oocyte Retrieval and embryo transfer

As detailed in our previous publication [25], we utilized specific methods for culturing oocytes and embryos. The decision between traditional IVF and ICSI was based on sperm quality. Post-fertilization, embryos were cultured until day 3 (D3) or day 5 (D5). The embryo transfer, limited to two embryos at a time, was performed under ultrasound guidance, either on D3 or D5 following oocyte collection. From the day after retrieval, participants received bi-daily 20 mg progesterone injections throughout the luteal phase. Guided by the policy of our center, only embryos at the D3 stage were transferred.

### Freeze-all strategy

In cases like high OHSS risk, thin endometrium, elevated progesterone levels, or personal preference, we employed the “freeze-all” approach. Criteria for this included age under 35, the use of hCG in ovarian stimulation, more than 20 follicles > 14 mm, or E2 levels above 5000 pg/ml on the trigger day, and insufficient endometrial thickness (< 7 mm).

### Exposure definitions

All participants had their blood samples collected before commencing IVF treatment. Fasting blood glucose (FBG), total cholesterol (TC), and triglyceride (TG) levels were measured using an autoanalyzer (AU 5800, Beckman Coulter). An exposure variable [17] was utilized as the TyG-BMI index, which was computed as the natural logarithm of [TG (mg/dL) × FBG (mg/dL)/2] × BMI [17].

### Outcome definitions

#### Clinical outcomes

We have defined the term ‘live birth’ as the act of giving birth to one or more infants who are alive and have reached a gestation period of at least 24 weeks. An elevated level of serum hCG (> 5 mIU/mL) was observed 14 days after embryo transfer, indicating a ‘biochemical pregnancy’ without the presence of a gestational sac confirmed by ultrasound. It was determined that a “clinical pregnancy” had been established 35 days after embryo transfer when an ultrasound detected a gestational sac with an observable fetal heartbeat. Early pregnancy losses [25] encompassed spontaneous miscarriage or ectopic pregnancy occurring prior to 12 weeks of gestation [26].

#### Laboratory outcomes

Our study measured several parameters related to oocytes and embryos. The parameters measured encompassed the overall count of oocytes collected, the number of two-pronucleus (2PN) oocytes, the quantity and rate of 2PN cleavage embryos, the count of viable embryos, the proportion of high-quality embryos, and the percentage of blastocyst formation. Using the Gardner Embryo/Blastocyst Grading System, which has three parts: a letter and two numbers, a morphologically “perfect” day 5 embryo transfer would be a 4AA; good expansion and excellent inner cell mass and trophectoderm. A grade 3 embryo may also be of good quality if its appearance can be explained by asynchronous cell division rather than by poor development. For day 3 embryos, the number of cells and the degree of fragmentation are the main criteria. An embryo that has 8 or more cells and less than 20% fragmentation is considered to have a high quality [27, 28].

### Statistical analysis

At the beginning of the study, the participants were categorized into four groups according to their TyG-BMI index quartiles. Mean ± standard deviation or median (interquartile range) were used to express continuous data, depending on distribution, whereas frequencies or percentages were used to represent categorical data. We employed One-Way ANOVA for data following a normal distribution, the Kruskal Wallis H test for skewed data, and the chi-square test for categorical variables to analyze differences in means and proportions. Univariate linear regression was used to evaluate the association between the TyG-BMI index and different outcomes. The results were presented in three models: unadjusted, minimally adjusted, and fully adjusted. In order to investigate the connections between the TyG-BMI index and the results of IVF/ICSI, we employed Generalized Additive Models (GAM) and Restricted cubic splines (RCS). Continuous confounders were incorporated into the RCS model with fixed points at the 5th, 50th, and 95th percentiles to analyze non-linear associations and dose-response relationships [29, 30]. For detailed analysis in univariate and multivariate models, the TyG-BMI index was normalized (Z-score) to evaluate the effect of each standard deviation increase on reproductive results. In addition, we performed subgroup analyses to investigate the impact of body mass index (BMI) and levels of Anti-Mullerian Hormone (AMH) on the relationship. Separately, the analysis was conducted on the interactions between the TyG-BMI index and these factors.A *P*-value of less than 0.05 was chosen as the threshold for statistical significance.R software (http //www.R-project.org, The R Foundation) and EmpowerStats (http//:www.empowerstats.com, X&Y Solution, Inc., Boston, MA) were utilized for conducting all statistical analyses.

## Results

### Baseline characteristics of study participants

**Fig. 1:**
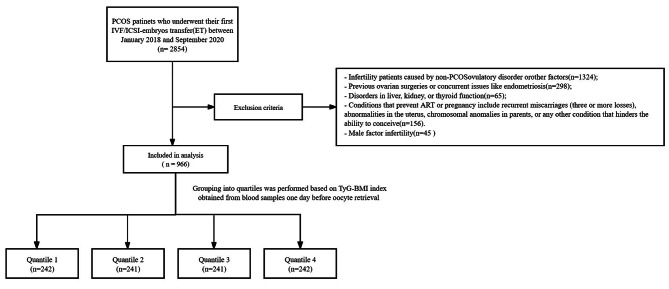
Flow-chart of the study cohort characteristics PCOS, Polycystic Ovary Syndorme; TyG-BMI, Triglyceride glucose-body mass

Figure 1 shows a flow chart of the study population. The TyG-BMI index of the study participants was categorized into four quartiles based on their baseline values (Quartile 1 (Q1): < 176.93, Quartile 2 (Q2): 176.93-203.19, Quartile 3 (Q3): 203.19-233.05, Quartile 4 (Q4): ≥ 233.05). The baseline characteristics of these groups are presented in Table 1, categorized based on their TyG-BMI index levels. Significantly lower BMI, shorter infertility duration, reduced Gn dosage, and lower progesterone levels on hCG administration day were observed in the Q1-Q3 groups, in contrast to the Q4 group with the highest TyG-BMI index.Significant variations were observed across the groups, showing an inverse relationship between the TyG-BMI index values and the levels of AMH and Antral Follicle Count (AFC).

**Table 1:** Baseline characteristics of participants by quartiles of TyG‑BMI index (*N* = 966)

Characteristics	Quantile 1	Quantile 2	Quantile 3	Quantile 4	P-value
N	242	241	241	242	
Age(year)	28.03 ± 2.98	28.38 ± 3.23	28.30 ± 3.25	28.53 ± 3.20	0.375
Primary infertility					0.317
No	70 (28.93%)	77 (31.95%)	62 (25.73%)	61 (25.21%)	
Yes	172 (71.07%)	164 (68.05%)	179 (74.27%)	181 (74.79%)	
Smoking history, n (%)					0.111
No	238 (98.35%)	241 (100.00%)	240 (99.59%)	241 (99.59%)	
Yes	4 (1.65%)	0 (0.00%)	1 (0.41%)	1 (0.41%)	
Duration of infertility(year)	2.00 (2.00–3.00)	3.00 (2.00–4.00)	3.00 (2.00–4.00)	3.00 (2.00–5.00)	0.615
BMI (kg/m^2^)					< 0.001
< 18.5	38 (15.70%)	0 (0.00%)	0 (0.00%)	0 (0.00%)	
≥ 18.5, < 25	204 (84.30%)	230 (95.44%)	109 (45.23%)	4 (1.65%)	
≥ 25, < 30	0 (0.00%)	11 (4.56%)	130 (53.94%)	141 (58.26%)	
≥ 30	0 (0.00%)	0 (0.00%)	2 (0.83%)	97 (40.08%)	
Basal FSH(IU/L)	6.98 ± 1.60	6.64 ± 1.73	6.43 ± 1.45	6.50 ± 2.93	0.016
Basal LH(IU/L)	7.29 ± 3.23	7.97 ± 3.08	7.10 ± 3.95	6.87 ± 3.91	0.243
Basal E2 (pg/mL)	44.00 (32.00-61.40)	45.00(33.00–58.00)	44.00(33.00–57.00)	42.00 (30.00–56.00)	0.531
Basla T (ng/dl)	0.54 (0.41–0.67)	0.55 (0.43–0.70)	0.57 (0.43–0.73)	0.59 (0.44–0.75)	0.981
AMH (ng/ml)	12.32 ± 5.33	11.54 ± 5.34	12.22 ± 5.40	10.34 ± 5.51	< 0.001
AFC	16.41 ± 5.14	16.30 ± 4.76	15.10 ± 4.83	14.12 ± 4.79	< 0.001
Starting dose of Gn (IU)	172.35 ± 28.65	180.39 ± 30.08	191.90 ± 32.86	213.34 ± 38.46	< 0.001
Total Gn dose (IU)	1479.62 ± 333.40	1643.59 ± 428.46	1900.37 ± 663.03	2345.38 ± 723.20	< 0.001
Duration of Gn (day)	8.69 ± 1.20	8.95 ± 1.41	9.32 ± 1.88	10.02 ± 2.03	< 0.001
Total GnRH-ant(IU)	1.12 (1.00-1.50)	1.25 (1.00-1.50)	1.00 (0.75–1.50)	1.12 (0.75–1.59)	0.139
Duration of GnRH-ant (day)	5.07 ± 1.85	5.12 ± 1.97	4.89 ± 1.85	5.19 ± 1.96)	0.341
Endometrial thickness on triggering day (mm)	9.67 ± 1.61	9.76 ± 1.54	9.88 ± 1.47	9.64 ± 1.56	0.241
E2 on triggering day (pg/ml)	7061.50 (5006.50-10079.00)	6566.00 (4870.00-9161.00)	5935.00 (4296.00-9087.00)	4856.00 (3433.50-7212.50)	< 0.001
P on triggering day (ng/ml)	1.47 (1.04–2.02)	1.36 (0.99–1.80)	1.30 (0.98–1.72)	1.14 (0.81–1.57)	< 0.001
LH on triggering day (IU/L)	2.38 (1.49–3.52)	2.81 (1.64–4.30)	2.62 (1.71–4.31)	3.03 (1.93–5.19)	0.020

Data are expressed as median (interquartile range) for non-normally distributed continuous variables

Data are expressed as mean + SD for normally distributed continuous variables

Categorical variables were expressed in frequency or as a percentage

Abbreviations: TyG-BMI index, triglyceride glucose-body mass index

BMI, body mass index; FSH, follicle-stimulating hormone; LH, luteinizing hormone; T, testosterone; AMH, anti-müllerian hormone; P, progesterone; AFC, antral follicle count; Gn, gonadotropin, GnRH-ant, gonadotropin releasing hormone antagonist, HCG, human chorionic gonadotropin

**Table 2:** ART outcome between the four groups

Characteristics	Quantile 1	Quantile 2	Quantile 3	Quantile 4	P-value
N	242	241	241	242	
No. of oocytes retrieved (n)	16.00 (12.00–20.00)	15.00 (12.00–19.00)	14.00 (11.00–19.00)	13.00 (9.00–16.00)	< 0.001
No. of 2PN cleavage (n)	13.00 (9.00–17.00)	12.00 (9.00–16.00)	11.00 (7.00–15.00)	10.00 (6.00–14.00)	< 0.001
No. of 2PN(n)(	13.00 (9.00-17.75)	13.00 (9.00–16.00)	11.00 (7.00–15.00)	10.00 (6.00–14.00)	< 0.001
Cleavage rate (%)	99.82 (3284/3290)	99.78 (3133/3140)	99.60 (2773/2784)	99.39 (2454/2469)	0.999
No. of cleavage embryos(n)	13.00 (9.25-18.00)	13.00 (9.00–16.00)	12.00 (7.00–15.00)	10.00 (6.00–14.00)	< 0.001
No. of available embryos(n)	8.00 (5.00–12.00)	8.00 (5.00–10.00)	6.00 (4.00–10.00)	6.00 (4.00–9.00)	< 0.001
Available embryo rate (%)	58.76 (607/1033)	58.58%(611/1043)	56.47%(467/827)	61.17(471/770)	0.814
High-quality embryos(n)	3.00 (1.00–7.00)	3.00 (1.00–5.00)	2.00 (1.00–5.00)	2.00 (0.00–4.00)	< 0.001
Blastocyst formation rate(%)	59.66(534/895)	55.51(509/917)	50.66(362/687)	58.41(361/618)	0.429
OHSS					0.002
No	216 (89.26%)	223 (92.53%)	232 (96.27%)	234 (96.69%)	
Yes	26 (10.74%)	18 (7.47%)	9 (3.73%)	8 (3.31%)	

Data are expressed as median (interquartile range) for non-normally distributed continuous variables

Data are expressed as mean + SD for normally distributed continuous variables

Categorical variables were expressed in frequency or as a percentage

Abbreviations: 2PN, two pronucleus; OHSS, ovarian hyperstimulation syndrome

When comparing the laboratory results, it was evident that there was a noticeable decline in the quantity of oocytes obtained as we transitioned from Q1 to Q4, as showed in Table 2. Patients in higher quantiles had a tendency to retrieve fewer oocytes, with the median count decreasing from 16.00 in Q1 to 13.00 in Q4. Likewise, there was an observable decline in the quantity of 2PN cleavage and the quantity of 2PN throughout the quantiles. Patients in the higher quantiles exhibited fewer 2PN cleavage and 2PN counts, signifying a potential decrease in fertility parameters as quantiles increased. Interestingly, while the cleavage rate showed a slight decrease across the quantiles, this variation was minimal. Patients from Q1 to Q4 had cleavage rates of 99.82–99.39% respectively, suggesting relatively consistent cleavage rates irrespective of quantile classification.

### Univariate analysis

The outcomes of the univariate analysis are detailed in Table 3 and Supplementary Table 1. The number of available embryos is positively correlated with AMH, basal LH, and AFC, as shown in Table 3. On the other hand, the TyG-BMI index, basal FSH, BMI, initial Gn dose, total Gn dose, and duration of Gn administration exhibited a negative correlation with the quantity of embryos that were available. The univariate analysis pertaining to high-quality embryos is provided in Supplementary Table 1.

**Table 3:** The results of uivariate analysis

No. of available embryos	Statistics	β(95%CI), P value
TyG-BMI index	207.69 ± 40.37	-0.02 (-0.03, -0.01) < 0.0001
Duration of infertility(year)	3.29 ± 2.05	-0.10 (-0.24, 0.03) 0.133
BMI (kg/m^2^)		
< 18.5	38 (3.93%)	0
18.5–25	547 (56.63%)	-0.35 (-1.78, 1.07) 0.626
25–30	282 (29.19%)	-1.39 (-2.86, 0.07) 0.063
≥ 30	99 (10.25%)	-2.71 (-4.33, -1.08) 0.001
AMH(ng/ml)	11.61 ± 5.45	0.14 (0.09, 0.19) < 0.0001
AFC	15.48 ± 4.96	0.40 (0.35, 0.45) < 0.0001
BASAL FSH (IU/L)	6.64 ± 2.02	-0.20 (-0.34, -0.06) 0.004
BASAL E2 (pg/ml)	49.07 ± 57.43	0.00 (-0.00, 0.01) 0.862
BASAL LH (IU/L)	7.63 ± 4.20	0.12 (0.06, 0.19) 0.0003
BASLA T (ng/dl)	0.75 ± 2.65	-0.02 (-0.12, 0.09) 0.757
Total Gn dose (IU)	1842.39 ± 648.48	-0.00 (-0.00, -0.00) < 0.0001
Starting dose of Gn (IU)	189.50 ± 36.14	-0.02 (-0.03, -0.01) < 0.0001
Duration of Gn (day)	9.25 ± 1.74	-0.21 (-0.37, -0.05) 0.011

Data is represented as β(95%CI), *P* value

Abbreviations: TyG-BMI index, triglyceride glucose-body mass index; BMI, body mass index;

FSH, follicle-stimulating hormone; LH, luteinizing hormone; T, testosterone;

AMH anti-müllerian hormone; AFC, antral follicle count; Gn, gonadotropin.

### The relationship between TyG-BMI index and laboratory outcomes

Table 4 presents the correlation between the quartiles of the TyG-BMI index and laboratory results.In our analysis, we utilized a univariate linear regression model to evaluate the connections between the quartiles of the TyG-BMI index and these outcomes. This assessment included both non-adjusted and adjusted models.

**Table 4:** Association between quartiles of TyG-BMI index and laboratory data among the whole participants (*N* = 966)

Variable	Crude model (β, 95%CI, P)	Minimally adjusted model (β, 95%CI, P)	Fully adjusted model (β, 95%CI, P)
No.of available embryos			
TyG-BMI index	-0.019 (-0.026, -0.012),<0.001	-0.003 (-0.016, 0.009), 0.583	-0.004 (-0.016, 0.008), 0.491
TyG-BMI index quartile			
Q1	Ref	Ref	Ref
Q2	-0.380 (-1.153, 0.393), 0.335	-0.197 (-0.937, 0.543), 0.602	-0.354 (-1.089, 0.380), 0.344
Q3	-1.496 (-2.269, -0.723),<0.001	-0.885 (-1.772, 0.003), 0.051	-0.949 (-1.840, -0.057), 0.037
Q4	-1.971 (-2.743, -1.199),<0.001	-0.313 (-1.543, 0.917), 0.617	-0.555 (-1.773, 0.663), 0.3714
*P* for trend	-0.703 (-0.947, -0.459),<0.001	-0.224 (-0.611, 0.163), 0.257	-0.285 (-0.671, 0.102), 0.149
Available embryo rate			
TyG-BMI index	0.000 (-0.000, 0.001), 0.228	0.001 (-0.000, 0.001), 0.058	0.000 (-0.000, 0.001), 0.241
TyG-BMI index quartile			
Q1	Ref	Ref	Ref
Q2	-0.001 (-0.042, 0.039), 0.945	0.003 (-0.039, 0.045), 0.887	-0.009 (-0.051, 0.033), 0.667
Q3	-0.012 (-0.053, 0.028), 0.549	-0.018 (-0.068, 0.032), 0.487	-0.027 (-0.079, 0.024), 0.296
Q4	0.024 (-0.016, 0.064), 0.242	0.054 (-0.016, 0.123), 0.129	0.023 (-0.047, 0.093), 0.521
*P* for trend	0.006 (-0.007, 0.019), 0.346	0.008 (-0.014, 0.030), 0.472	0.000 (-0.022, 0.022), 0.993
High-quality embryos			
TyG-BMI index	-0.013 (-0.018, -0.007),<0.001	-0.006 (-0.016, 0.004), 0.251	-0.005 (-0.015, 0.004), 0.274
TyG-BMI index quartile			
Q1	Ref	Ref	Ref
Q2	-0.870 (-1.474, -0.267), 0.004	-0.824 (-1.427, -0.221), 0.007	-0.843 (-1.437, -0.249), 0.005
Q3	-1.144 (-1.748, -0.540),<0.001	-0.833 (-1.557, -0.110), 0.024	-0.829 (-1.550, -0.108), 0.024
Q4	-1.579 (-2.181, -0.976),<0.001	-0.817 (-1.819, 0.185), 0.111	-0.832 (-1.816, 0.153), 0.098
*P* for trend	-0.501 (-0.692, -0.310),<0.001	-0.352 (-0.667, -0.037), 0.028	-0.349 (-0.662, -0.036), 0.028

Tests for linear trend were conducted by assigning median values of each quartile of TyG-BMI index as a continuous variable in the models

Crude model: did not adjust other covariants

Minimally adjusted model: adjusted age; AMH; BMI; AFC

Fully adjusted model: adjusted age; Basal FSH; Basal E2; Basal LH; AMH; AFC; Total Gn dose; Duration of Gn;

E2 on triggering day; P on triggering day. CI, confidence interval; Ref, reference

Abbreviations: TyG-BMI index, triglyceride glucose-body mass index

In the crude model, the TyG-BMI index demonstrated no significant link with the proportion of usable embryos. Nonetheless, an inverse relationship was noted with both the quantity of usable embryos and the number of embryos of superior quality. In the model with minimal adjustments, which took into account age, AMH, BMI, and AFC, the higher quartiles of the TyG-BMI index exhibited inverse associations with the quantity of usable embryos (β = -0.224, 95%, trend *P*-value = 0.257) and the count of high-quality embryos (β = -0.352, 95%, trend *P*-value = 0.028), when compared to the lowest quartile.

No significant correlations were observed between the quartiles of the TyG-BMI index and the proportion of usable embryos. This trend persisted in the fully adjusted model, which also included variables such as baseline FSH, E2, LH levels, AMH, AFC, total Gn dosage, duration of Gn administration, E2 levels on the day of hCG administration, and Progesterone levels on the day of hCG administration.

### The analyses of non-linear relationship

**Fig. 2:**
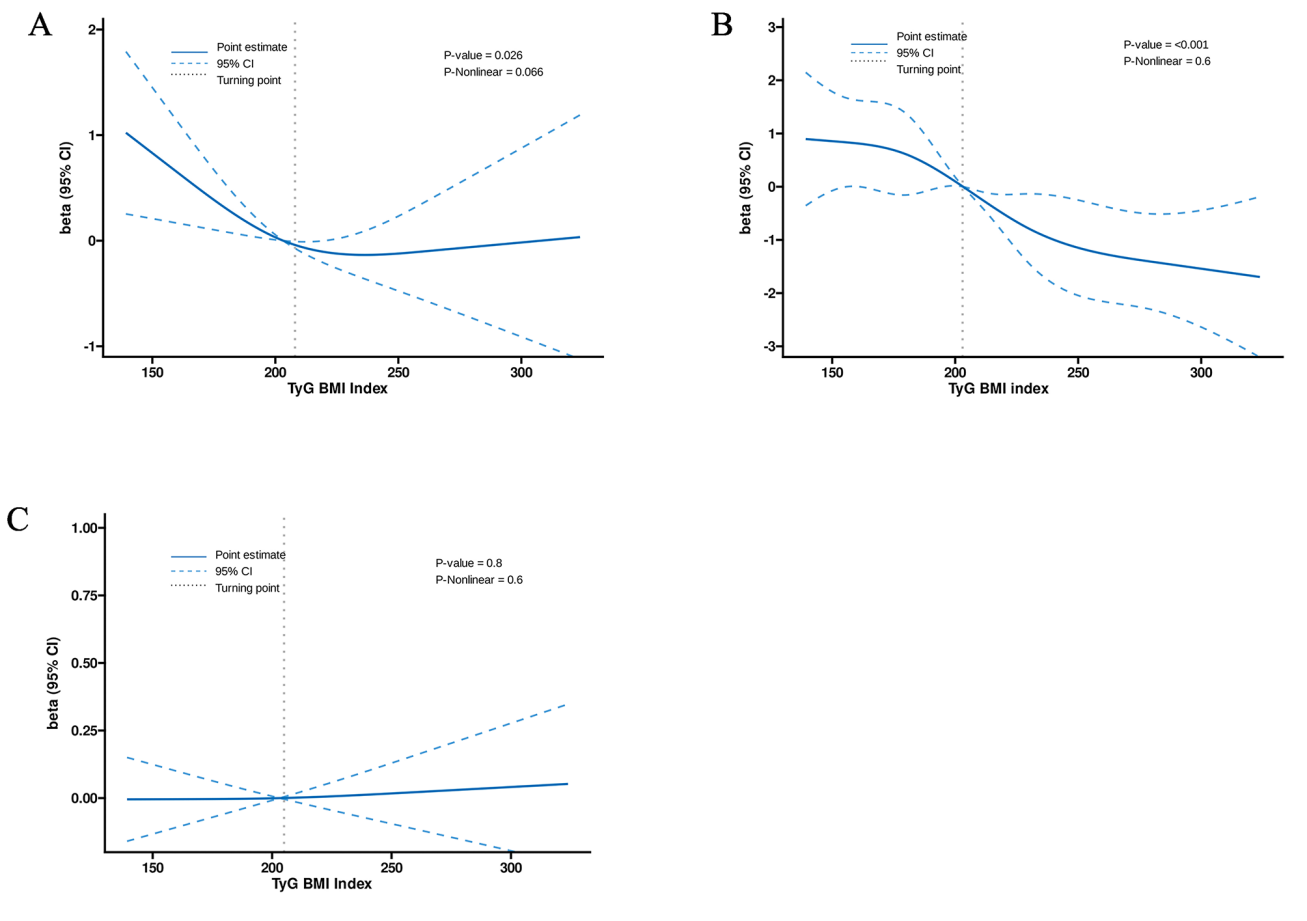
Association between TyG-BMI index and reproductive outcomes. (**A**) High quality embryos. (**B**) No. of available embryos. (**C**) Available embryo rate. The models were adjusted for age, duration of infertility, BMI, basal FSH, AMH, starting and total dose of Gn, duration and total dose of GnRH-ant, endometrial thickness on trigger day, E2 and P on HCG start day

We examined the correlation between the TyG-BMI index and different outcomes in our research, considering that the TyG-BMI index is a continuous variable, as shown in Fig. 2.To define this non-linear relationship, we established RCS models for the TyG-BMI index (per 1 unit increase).

According to the RCS models, there was a direct correlation observed between the TyG-BMI index and the quantity of embryos accessible (*P*-non-linear = 0.6, *P*-overall < 0.001), as well as with the number of superior embryos (*P*-nonlinear = 0.026, *P*-overall = 0.006). Nevertheless, based on the RCS model, there was no substantial correlation observed between the TyG-BMI index and the rate of accessible embryos (*P*-nonlinear = 0.60, *P*-overall = 0.8).

### Univariable and Multivariate regression analysis of pregnancy outcomes

The findings in Table 5 indicate that neither univariate nor multivariate logistic regression analyses revealed any significant associations between different quartiles of the TyG-BMI index and various reproductive outcomes.The results encompass the rate of biochemical pregnancy (BPR), rate of clinical pregnancy (CPR), rate of live birth (LBR), and rate of early pregnancy loss (EPLR).

**Table 5:** Univariable and Multivariate regression analysis of pregnancy outcomes among the whole participants

Variable	Outcome	Univariate analysis		Multivariate analysis	
		OR	P	OR	P
Biochemical pregnancy rate (%)					
TyG-BMI index quartile					
Q1	66.66(6/9)	Ref		Ref	
Q2	60.00(6/10)	0.75 (0.11, 4.94)	0.764	0.11 (0.01, 1.45)	0.136
Q3	80.00(8/10)	2.00(0.25, 19.18)	0.513	1.13 (0.83, 1.67)	0.466
Q4	50.00(5/10)	0.50 (0.07, 3.14)	0.465	2.40 (0.11, 104.89)	0.602
Clinical pregnancy rate (n%)					
TyG-BMI index quartile					
Q1	55.56(5/9)	Ref		Ref	
Q2	60.00(6/10)	1.20 (0.19, 7.44)	0.845	0.30 (0.02, 3.11)	0.334
Q3	60.00(6/10)	3.20 (0.42, 24.42)	0.262	0.75 (0.45, 1.12)	0.195
Q4	50.00(5/10)	0.80 (0.13,4.87)	0.809	2.70 (0.17,91.08)	0.509
Early pregnancy loss rate (n%)					
TyG-BMI index quartile					
Q1	0.00(0/6)	Ref		Ref	
Q2	16.67(1/6)	-	0.296	-	-
Q3	12.5(1/8)	-	0.369	-	-
Q4	20.00(1/5)	-	-	-	-
Live birth rate (n%)					
TyG-BMI index quartile					
Q1	44.44(4/9)	Ref		Ref	
Q2	50(5/10)	1.25 (0.205, 7.615)	0.809	0.689 (0.636, 0.069)	0.689
Q3	50(5/10)	1.25 (0.205, 7.615)	0.809	1.432 (0.186, 11.058)	0.730
Q4	40(4/10)	0.91 (0.205, 7.615)	0.742	3.319 (0.216, 10.598)	0.389

All values are ORs (95% CIs). Values were determined by using logistic regression. OR, odds ratio;

*P* value shows significance of entrance in the logistic regression model; *P* values in bold indicate statistical significance

### Subgroup analysis

To evaluate the impact of potential confounding factors, such as AMH and BMI, subgroup analyses were performed as outlined in Table 6.Significant interactions were observed between the subgroups categorized by AMH levels and the influence of the TyG-BMI index on the quantity of accessible embryos (P-value for interaction = 0.012). Supplementary Tables 2 and 3 also demonstrated comparable results concerning the impact of the TyG-BMI index on the rates of available embryos and high-quality embryos.

**Table 6:** Subgroup analysis for association between TyG-BMI index (per 1 SD) and No. of available embryos

Subgroup	n total	Unadjusted β (95% CI)	Unadjusted P value	Adjusted β (95% CI)	Adjusted P value	P for interaction
BMI (kg/m^2^)						0.215
< 18.5	38	1.965 (-3.099, 7.029)	0.452	1.123 (-3.003, 5.249)	0.598	
≥ 18.5, < 25	547	-0.836 (-1.596, -0.0077)	0.031	-0.411 (-1.128, 0.306)	0.262	
≥ 25, < 30	282	-0.022 (-0.968, 0.925)	0.964	0.402 (-0.474, 1.278)	0.369	
≥ 30	99	-0.060 (-1.115, 0.996)	0.912	0.391 (-0.480, 1.261)	0.381	
AMH (ng/ml)						0.012
Low	311	-0.298 (-0.706, 0.111)	0.155	0.304 (-0.168, 0.775)	0.208	
Middle	313	-0.524 (-0.996, -0.051)	0.031	-0.038 (-0.585, 0.509)	0.892	
High	312	-1.273 (-1.850, -0.695)	< 0.0001	-1.090 (-1.705, -0.476)	< 0.001	

Abbreviations: BMI, body mass index; AMH, anti-müllerian hormone

## Discussion

Our research examined the correlation between the TyG-BMI indicator and females diagnosed with PCOS who are receiving treatment through the GnRH-ant protocol. The key outcomes revealed that higher TyG-BMI index values were associated with less favorable ovarian responses. This was evidenced by a lower yield of retrieved oocytes, a decreased number of 2PN embryos, and compromised embryo quality. However, there was no notable association found between the TyG-BMI index and pregnancy or live birth results, possibly due to the limited availability of fresh embryo transfer data. While prior studies have linked various metabolic parameters to reproductive outcomes [31–33], this research represents the first analysis specifically examining the predictive utility of TyG-BMI for IVF success in a PCOS population. Our study indicates that TyG-BMI, similar to its role in cardiovascular research, could potentially be used as a predictive indicator for reproductive outcomes in addition to its significance in predicting cardiovascular events in CAD patients.

Many PCOS patients exhibit metabolic abnormalities, including IR, impaired glucose tolerance, a higher risk of developing type 2 diabetes mellitus (T2DM), and factors that make them more susceptible to cardiovascular diseases [34]. There is a notable overlap in the pathophysiological and clinical features of PCOS and MetS, with MetS and its individual elements often present in those with PCOS [35]. The prevalence of MetS in individuals with PCOS is said to be approximately 43%, which is almost twice as high as the rate observed in females of similar age in the overall population.In particular, there were 50 individuals diagnosed with MetS, 23 displayed a single metabolic abnormality, and 12 presented with two metabolic irregularities. Consequently, 85 patients, or 63.4% of the PCOS group, showed varying degrees of metabolic issues [36]. Dyslipidemia, a critical aspect of MetS, is prevalent in approximately 70% of PCOS patients [37]. A substantial amount of evidence indicates that insulin resistance is a notable contributing factor to various conditions including type 2 diabetes mellitus, lipid disorders, excessive weight, and heart diseases [38]. The lipoprotein profile is significantly modified by IR, resulting in changes to the amount and characteristics of TG, TC transport, and lipoprotein oxidation. These alterations influence the development of atherosclerosis [39]. In relation to metabolic irregularities, numerous women with PCOS display traits that are consistent with metabolic syndrome, including elevated levels of TG, total TC, and low density lipoprotein cholesterol (LDL-C), as well as decreased levels of high density lipoprotein cholesterol (HDL-C) [40]. Prior studies have demonstrated that IR is linked to the advancement of cardiovascular diseases and can predict cardiovascular outcomes [41]. Consequently, the TyG index was developed as a tool to assess IR. Additionally, BMI is widely recognized as a measure of obesity and an indicator of IR. Professor Er and colleagues introduced the TyG-BMI index in 2016, which is a combination of the TyG index and BMI [17]. It was discovered in a study that TyG-BMI was a more accurate indicator of insulin resistance compared to TyG [16]. Studies have indicated that the TyG-BMI indicator is superior to the TyG indicator alone when it comes to identifying NAFLD in non-obese patients [42]. According to recent studies, the TyG-BMI indicator outperforms the TyG indicator in accurately detecting metabolic syndrome among individuals with PCOS [19]. The discovery implies that the TyG-BMI index can serve as a holistic indicator for overall health problems.

Our study found that higher TyG-BMI quartiles were linked to a reduced quantity and quality of embryos, supporting the idea that metabolic disruptions can have negative impacts on reproductive processes.This parallel between cardiovascular and reproductive outcomes underscores the systemic impact of metabolic dysregulation. Further investigation is needed to explore the underlying mechanisms that determine the association between the TyG-BMI index and reproductive outcomes in women diagnosed with PCOS. This association can be explained in various ways.Firstly, IR is regarded as the core mechanism of PCOS pathogenesis, IR and hyperinsulinemia are common features of PCOS and MetS, and they can impair the ovarian function and the oocyte quality in several ways.First, hyperinsulinemia can stimulate the ovarian theca cells to produce more androgens, which can inhibit the follicular development and the aromatization of androgens to estrogens [43]. Furthermore, excessive insulin levels can decrease the synthesis of sex hormone-binding globulin (SHBG), leading to elevated levels of unbound androgens and estrogens [44]. Also, hyperinsulinemia has the potential to disrupt the regulation of the hypothalamic-pituitary-ovarian (HPO) axis, resulting in elevated LH secretion and reduced FSH secretion. This disturbance can negatively impact follicular maturation and ovulation [40]. Fourth, hyperinsulinemia may induce oxidative stress, inflammation, and apoptosis in the ovarian granulosa cells and the oocytes, which can affect the oocyte quality and the embryo development [45]. As TyG-BMI elevations reflect worsening insulin resistance, this provides a plausible Biological link between heightened TyG-BMI and suboptimal embryo yields. The ovotoxic effects of inflammation provide another pathway explaining the TyG-BMI and IVF outcome relationship. Systemic inflammation promotes ovarian oxidative damage which may diminish oocyte quality and embryo competence [46, 47]. Since TyG-BMI tracks with inflammatory burden, greater inflammation among high TyG-BMI females may jeopardize IVF success.

Furthermore, a heightened TyG index indicates more than just insulin resistance; it also indicates a variety of negative health conditions such as cerebrovascular disease, cardiovascular disease, obesity, diabetes, hypertension, metabolic syndrome, and disorders in lipid metabolism. Furthermore, the significance of inflammation and oxidative stress plays a vital part in connecting the TyG-BMI index with PCOS.A high TyG index is associated with endothelial dysfunction, inflammatory responses, and oxidative stress. Inflammation can impair vascular endothelium, and oxidative stress may similarly damage vascular endothelium, potentially impacting the quality of oocytes and embryos.

PCOS, which is a diverse condition with hormonal imbalances and metabolic issues, impacts multiple aspects of reproduction such as follicle development and embryo implantation [48]. Previous studies have linked PCOS with negative impacts on ovarian response and outcomes of IVF, as well as an increased likelihood of miscarriages [49]. Our multivariate logistic regression analysis did not demonstrate that higher TYG-BMI was negatively associated with BPR, clinical CPR, or LBR. However, the limited quantity of fresh embryo transfers (only 49 out of 966 IVF cycles) restricts interpretability, as most women underwent freeze-all and their later frozen ET outcomes were not tracked. Furthermore, specific details on subsequent frozen ETs, cumulative pregnancy rates accounting for additional frozen cycles, and live births were not adequately recorded. These data constraints preclude sufficiently powered analysis of correlations between TyG-BMI and actual reproductive success… Hence we have refocused this report exclusively on impacts of TyG-BMI on ovarian responses and oocyte/embryo parameters, rather than on underpowered clinical endpoints. Additional investigation is necessary to validate our initial discoveries by conducting studies with larger sample sizes of newly implanted embryos. Notably, there was an intricate interplay observed between the TyG-BMI index and the quantity of high-quality embryos, underscoring the complex connection between MetS and fertility results in individuals with PCOS.

Our research has several strengths. Significantly, this is the initial observational study that reveals the correlation between the TyG-BMI indicator and the outcomes of ARTin females diagnosed with PCOS. Additionally, we accounted for various confounding factors to ensure more reliable results. Nevertheless, it is important to take into account certain restrictions.Because our study is observational and conducted in a single center, we are unable to establish causality. Furthermore, measurements of triglycerides, glucose, and other parameters were only taken at the beginning of the study and could have potentially fluctuated throughout the follow-up period as a result of alterations in the participants’ lifestyles and medication usage. Third, the frequency of fresh embryo transfers in PCOS patients was lower, attributed to their higher risk of ovarian hyperstimulation syndrome. Moreover, other variables like therapies for metabolic syndrome, eating patterns, and levels of physical exercise were not taken into consideration. Consequently, future prospective studies with larger sample sizes are needed to better establish causality.

## Conclusions

According to our study, there is a connection between higher TyG-BMI levels and less favorable reproductive results in women with PCOS. While the TyG-BMI’s role in cardiovascular prognosis is gaining acceptance, its place in reproductive health is still being charted. Further research should aim to unravel the complex interactions between metabolic health and reproductive success, potentially improving IVF outcomes through targeted metabolic interventions.

### Electronic supplementary material

Below is the link to the electronic supplementary material.

Supplementary Material 1

## Data Availability

No datasets were generated or analysed during the current study.
