# Supplementary material for: The association between triglyceride glucose-body Mass Index and in vitro fertilization outcomes in women with polycystic ovary syndrome: a cohort study
Source: J Ovarian Res. 2024 Apr 26;17:90. doi: 10.1186/s13048-024-01416-1 (PMC11055342; doi:10.1186/s13048-024-01416-1)
Supplement: Supplementary file 1 — Supplementary Material 1 [file 13048_2024_1416_MOESM1_ESM.docx]

Supplementary Table

Supplementary Table1 The results of uivariate analysis

| High-quality embryos | Statistics | β(95%CI), *P* value |
| --- | --- | --- |
| TyG-BMI index | 207.69 ± 40.37 | -0.01 (-0.02, -0.01) <0.0001 |
| Duration of infertility(y) | 3.29 ± 2.05 | -0.02 (-0.12, 0.09) 0.729 |
| BMI (kg/m^2^) |  |  |
| <18.5 | 38 (3.93%) | 0 |
| 18.5-25 | 547 (56.63%) | -0.57 (-1.69, 0.54) 0.315 |
| 25-30 | 282 (29.19%) | -1.20 (-2.35, -0.04) 0.042 |
| ≥30 | 99 (10.25%) | -1.93 (-3.20, -0.66) 0.003 |
| AMH(ng/ml) | 11.61 ± 5.45 | 0.08 (0.04, 0.12) <0.0001 |
| AFC | 15.48 ± 4.96 | 0.26 (0.22, 0.31) <0.0001 |
| BASAL.FSH (IU/L) | 6.64 ± 2.02 | -0.11 (-0.22, -0.00) 0.045 |
| BASAL.E2 (pg/ml) | 49.07 ± 57.43 | 0.00 (-0.00, 0.00) 0.946 |
| BASAL.LH (IU/L) | 7.63 ± 4.20 | 0.07 (0.02, 0.12) 0.007 |
| BASLA.T (ng/dl) | 0.75 ± 2.65 | -0.02 (-0.10, 0.06) 0.578 |
| Total Gn dose (IU) | 1842.39 ± 648.48 | -0.00 (-0.00, -0.00) <0.0001 |
| Starting dose of Gn (IU) | 189.50 ± 36.14 | -0.01 (-0.02, -0.00) 0.0007 |
| Duration of Gn (day) | 9.25 ± 1.74 | -0.15 (-0.28, -0.03) 0.016 |

Data is represented as β(95%CI), P value.

Abbreviations：TyG-BMI index,triglyceride glucose-body mass index; BMI, body mass index;

FSH, follicle-stimulating hormone; LH, luteinizing hormone; T, testosterone;

AMH anti-müllerian hormone; AFC,antral follicle count; Gn, gonadotropin.

Supplementary Table2 Subgroup analysis for association between TyG-BMI index (per 1 SD) and available embryo rate

| Subgroup | n total | Unadjusted  β (95% CI) | Unadjusted  P value | Adjusted  β(95% CI) | Adjusted  *P* value | *P* for interaction |
| --- | --- | --- | --- | --- | --- | --- |
| BMI(kg/m^2^) |  |  |  |  |  | 0.272 |
| <18.5 | 38 | 0.149 (-0.102, 0.401) | 0.253 | 0.122 (-0.137,0.382) | 0.363 |  |
| ≥18.5, <25 | 547 | -0.002 (-0.041, 0.037) | 0.928 | -0.014 (-0.0055, 0.028) | 0.522 |  |
| ≥25, <30 | 282 | 0.054 (-0.001, 0.109) | 0.056 | 0.049 (-0.007, 0.105) | 0.089 |  |
| ≥30 | 99 | 0.047 (-0.025, 0.119) | 0.200 | 0.018 (-0.057, 0.092) | 0.646 |  |
| AMH |  |  |  |  |  | 0.201 |
| Low | 311 | 0.031 (0.005, 0.056) | 0.018 | 0.029 (-0.004, 0.063) | 0.085 |  |
| Middle | 313 | 0.015 (-0.011, 0.040) | 0.263 | -0.001 (-0.033, 0.032) | 0.969 |  |
| High | 312 | -0.007 (-0.035, 0.021) | 0.639 | -0.042 (-0.073, -0.011) | 0.009 |  |

Abbreviations：BMI, body mass index; AMH, anti-müllerian hormone

Supplementary Table3 Subgroup analysis for association between TyG-BMI index (per 1 SD) and High-quality embryos

| Subgroup | n total | Unadjusted  β(95% CI) | Unadjusted  *P* value | Adjusted  β(95% CI) | Adjusted  *P* value | *P* for interaction |
| --- | --- | --- | --- | --- | --- | --- |
| BMI |  |  |  |  |  | 0.022 |
| <18.5 | 38 | 2.792 (-1.402, 6.986) | 0.2003 | 2.138 (-1.628, 5.905) | 0.2744 |  |
| ≥18.5, <25 | 547 | -0.889 (-1.481, -0.297) | 0.0034 | -0.435 (-1.012, 0.143) | 0.1404 |  |
| ≥25, <30 | 282 | -0.001 (-0.749, 0.747) | 0.9974 | 0.212 (-0.511, 0.935) | 0.5656 |  |
| ≥30 | 99 | 0.175 (-0.595, 0.946) | 0.6566 | 0.340 (-0.469, 1.149) | 0.4119 |  |
| AMH |  |  |  |  |  | 0.016 |
| Low | 311 | -0.242 (-0.565, 0.081) | 0.7197 | 0.298 (-0.079, 0.675) | 0.1227 |  |
| Middle | 313 | -0.322 (-0.690, 0.046) | 0.0876 | -0.053 (-0.502, 0.396) | 0.8175 |  |
| High | 312 | -0.973 (-1.441, -0.505) | <0.0001 | -0.582 (-1.092, -0.073) | 0.0259 |  |

## Abbreviations：BMI, body mass index; AMH, anti-müllerian hormone
